# Size Dependency of Circulation and Biodistribution of Biomimetic Nanoparticles: Red Blood Cell Membrane-Coated Nanoparticles

**DOI:** 10.3390/cells8080881

**Published:** 2019-08-13

**Authors:** Haichun Li, Kai Jin, Man Luo, Xuejun Wang, Xiaowen Zhu, Xianping Liu, Ting Jiang, Qin Zhang, Sheng Wang, Zhiqing Pang

**Affiliations:** 1 Department of Colorectal Surgery, Fudan University Shanghai Cancer Center, Shanghai 200032, China; 2 School of Pharmacy, Fudan University, Key Laboratory of Smart Drug Delivery, Ministry of Education, 826 Zhangheng Road, Shanghai 201203, China; 3 Department of Bioengineering, School of Engineering and Applied Sciences, University of Pennsylvania, Philadelphia, PA 19104, USA; 4 School of Pharmacy, Heilongjiang University of Chinese Medicine, 24 Heping Road, Harbin 150040, China; 5 Department of Radiation Oncology, Shanghai Chest Hospital, Shanghai Jiao Tong University, Shanghai 200030, China

**Keywords:** biomimetic nanoparticles, cell membrane-coated nanoparticles, particle size, circulation, biodistribution

## Abstract

Recently, biomimetic nanoparticles, especially cell membrane-cloaked nanoparticles, have attracted increasing attention in biomedical applications, including antitumor therapy, detoxification, and immune modulation, by imitating the structure and the function of biological systems such as long circulation life in the blood. However, the circulation time of cell membrane-cloaked nanoparticles is far less than that of the original cells, greatly limiting their biomedical applications, while the underlying reasons are seldom demonstrated. In this study, the influence of particle size on the circulation and the biodistribution of red blood cell membrane-coated nanoparticles (RBC-NPs) as model biomimetic nanoparticles were investigated. Differently sized RBC-NPs (80, 120, 160, and 200 nm) were prepared by fusing RBC membranes on poly(lactic-co-glycolic acid) nanoparticles. It was shown that the particle size did not change the cellular uptake of these biomimetic nanoparticles by macrophage cells in vitro and their immunogenic responses in vivo. However, their circulation life in vivo decreased with the particle size, while their accumulation in the liver increased with the particle size, which might be related to their size-dependent filtration through hepatic sinusoids. These findings will provide experimental evidence for the design and the optimization of biomimetic nanoparticles.

## 1. Introduction

In the past two decades, nanoparticles ranging from 1 to 1000 nm have been extensively exploited in drug delivery, as they have some great advantages, such as the properties of tissue selectivity, long circulation, protection of encapsulated drugs, enhancing drug absorption and bioavailability, reducing renal clearance, biodegradability, biocompatibility, and even a prolonged pharmacological effect [1]. However, the in vivo pharmacokinetics of most conventional nanoparticles including PEGylated nanoparticles is still unsatisfactory and even gets worse after repeated administrations [2,3,4], which greatly limits their translation. An important reason is that the sophisticated immune system in the body could recognize the exogenous nanoparticles as invaders and eliminate them quickly [5].

Natural red blood cells (RBCs) are homologous with the autologous immune cells and will not be cleared by the immune systems until they are damaged or dead [6]. Therefore, RBCs can circulate in the blood for up to 120 days. This inspires coating RBC membranes or other cell membranes to nanoparticles, camouflaging them in vivo and making them escape from the monitoring of the immune system. Studies have shown that RBC membranes with abundant self-markers can help the nanoparticles to escape from the recognition of the immune system, greatly extending the circulation half-life of poly(lactic-co-glycolic acid) (PLGA) nanoparticles from a few hours to approximately 40 h [7,8,9,10]. Our studies also demonstrated that RBC membrane-coated melanin, polypyrrole nanoparticles, or gold nanorods exhibited a longer blood circulation time and a higher tumor accumulation compared with bare melanin, polypyrrole nanoparticles, or gold nanorods [11,12,13]. As a promising drug delivery system, cell membrane-coated nanoparticles have attracted increasing attention from researchers worldwide. These biomimetic nanoparticles combine the advantages of both synthetic nanoparticles and natural cell membranes with surface antigenic diversity, making precise drug delivery possible [6,14]. For instance, the macrophage cell membrane coating not only confers gold nanoshells or PLGA nanoparticles with the property of long circulation but also enhances their tumor or arthritis targeting ability through the identification function of macrophage membranes [15,16,17]. Cancer cell membrane-coated nanoparticles demonstrate a specific homologous targeting ability to cancer cells in vitro and in vivo [18,19,20]. Platelet membrane-coated nanoparticles show enhanced targeting capabilities to injury vessels [21], rheumatoid arthritis [22], atherosclerotic plaques [23,24], bacteria [21], and tumor cells [25]. Although cell membrane-coated nanoparticles have made great progress in drug delivery, detoxification [26,27,28,29], and vaccination [30,31,32], their half-life in vivo ranges from several hours to 40 h, which is still far less than that of the original natural cells. However, little is known about what results in a shorter circulation time of these biomimetic nanoparticles.

For conventional nanoparticles, their circulation time, cellular uptake, and in vivo tissue distribution are mainly controlled by various factors, such as diameter, shape, surface charge, and rigidness [1,33,34]. For instance, micro-sized, long, flexible filomicelles could not be internalized by macrophages and have a very long circulation of days or even weeks [35]. Compared with nanospheres, nanorods have a better affinity with cells and can penetrate tumors more rapidly, thus accumulating more in tumors, because the elongated shape has a larger surface area and higher transmembrane transport and diffusion rates than the spherical shape with the same diameter [36]. Studies have shown that negatively charged gold nanoparticles can accumulate in the liver more easily than positively charged gold nanoparticles [37]. As commonly known, the diameter is a key factor to nanoparticles, which can influence their in vivo process. Nanoparticles larger than 200 nm are more likely to be recognized by the immune systems and cleared by the liver and the spleen, while nanoparticles smaller than 5 nm are more likely to be filtered by the kidney [38]. Although much work has been done to elucidate the mechanisms underlying the short circulation and the specific biodistribution of conventional nanoparticles, little has been done to uncover the factors determining the in vivo life of biomimetic nanoparticles, even though their in vivo circulation and biodistribution are quite different from conventional nanoparticles.

The tight junction between capillary endothelial cells is the main barrier that prevents foreign substances or nanoparticles in the blood from entering tissues. However, the capillary endothelial cells in some tissues such as the liver, the spleen, the bone marrow, and tumors are discontinuous and have a lot of fenestraes [39], which could facilitate nanoparticles with a certain size to pass through this discontinuous endothelial structure or trap them in these tissues [40]. Thus, we conjecture that the particle size is one of the important factors determining the fate of biomimetic nanoparticles in vivo, but few studies have reported the role of particle size in their circulation and biodistribution in the body. In this study, RBC membrane-coated nanoparticles (RBC-NPs) are selected as the model biomimetic nanoparticles, since they have good biocompatibility, biodegradability, and long circulation in the bloodstream [41]. Differently sized RBC-NPs (80, 120, 160, and 200 nm) were prepared and characterized. The influences of particle size on cellular uptake by the macrophages, filtration through syringe filters with nanosized pores, circulation, biodistribution, and immunological response of RBC-NPs were investigated to find out some clues determining the in vivo life of biomimetic nanoparticles and provide experimental evidence for the design and the optimization of biomimetic nanoparticles.

## 2. Materials and Methods

### 2.1. Materials

Carboxyl group-terminated 50:50 poly(lactic-co-glycolic acid) (PLGA-COOH) was purchased from Lactel Absorbable Polymers (Pelham, AL, USA), and 1,1′-dioctadecyl-3,3,3′,3′–tetramethylindodicarbocyanine 4-chlorobenzenesulfonate salt (DiD) was obtained from Fanbo Biochemical Co., Ltd. (Beijing, China). Coumarin-6 was purchased from Sigma (Saint Louis, MO, USA). IgM and IgG ELISA kit and 4′,6-diamidino-2-phenylindole (DAPI) were purchased from Beyotime Biotechnology (Nantong, China), and 10% Sodium dodecyl sulfate-polyacrylamide gel was ordered from Biorad (Hercules, CA, USA). NuPAGE^®^ LDS sample buffer was purchased from Invitrogen (Carlsbad, CA, USA). Nitrocellulose membranes were purchased from PALL (Port Washington, NY,, USA). CD47 rabbit polyclonal primary antibody was obtained from Abcam (Cambs, UK). CD31 goat polyclonal primary antibody was purchased from R&D (Minneapolis, MN, USA). Alexa Fluor^®^ 488-conjugated donkey-goat secondary antibody and horseradish peroxidase (HRP)-conjugated donkey-rabbit secondary antibody were purchased from Jackson (West Grove, PA, USA). Immobilon™ Western Chemiluminescent HRP Substrate was obtained from Millipore (Billerica, MA, USA). Dulbecco’s modified Eagle’s medium (DMEM) and fetal bovine serum (FBS) were purchased from Life Technologies (Carlsbad, CA, USA). RAW 264.7 cells were obtained from ATCC (Gaithersburg, MD, USA). Deionized water was produced from Milli-Q Integral (Merck, Germany). All other reagents were purchased from Sinopharm Chemical Reagent Co. Ltd. (Shanghai, China).

Male ICR mice aged six weeks were purchased from Slac Lab Animal Co., Ltd. (Shanghai, China). All animal experiments were performed according to the protocols approved by the Animal Experiment Ethics Committee of Fudan University (2014-03-YJ-PZQ-01).

### 2.2. RBC Ghost Deviation

The RBC membrane was derived from the whole blood as previously described [7]. Briefly, the whole blood was collected from ICR mice aged six weeks (SLAC Lab. Animal, Shanghai, China) by cheek pouch puncture. The whole blood was centrifuged at 700× *g* for 10 min at 4 °C to remove the plasma, white blood cells, and platelets. The resulting RBCs were washed three times with ice-cold 1×PBS containing 1 mM EDTA∙2Na. Then the purifyied RBCs were ruptured in 0.2 mM EDTA∙2Na solution and centrifuged at 20,000× *g* for 10 min at 4 °C. The supernatant was discarded, and the collected pellet was further purified with 0.2 mM EDTA∙2Na solution. The resulting RBC ghost was collected and stored at −80 °C for further use.

### 2.3. Preparation of RBC-NPs with Different-Sized Polymeric Cores

Polymeric nanoparticles with different particle sizes were prepared by the nanoprecipitation method [42]. Briefly, 1 mL of PLGA acetone solution (4, 10, and 20 mg/mL) was injected into 2 mL of deionized water. The mixture was evaporated in a vacuum chamber for 2 h to remove the acetone, and PLGA nanoparticle cores of around 60 nm, 100 nm, and 140 nm were finally obtained. To prepare PLGA nanoparticle cores of around 180 nm, 2 mL of water was dripped into 1 mL of PLGA acetone solution (20 mg/mL), and the acetone was removed as described above. For the preparation of RBC-NPs, the appropriate amount of RBC ghost, which was calculated according to the nanoparticle surface area (Table 1), was added into different-sized PLGA nanoparticle solutions, assuming that the surface area of each RBC [43] was 75 μm^2^, and the density of the PLGA was 1.2 mg/mL [28]. The number of RBCs in 1 mL of the blood in mammals [11] was reported as ~5 × 10^9^. RBC membranes were fused on the surface of PLGA nanoparticles by ultrasonic treatment for 2 min using a bath sonicator (SG5200HE, Gutel, China). DiD or coumarin-6 dye-labeled RBC-NPs were prepared by the same method as described above, except that DiD or coumarin-6 (0.1% of PLGA mass) was dissolved in the polymer solution.

### 2.4. Characterization of RBC-NPs

The size and zeta potential of RBC-NPs were measured using a dynamic light scattering (DLS) detector (Zetasizer, Nano ZS, Malvern, UK). The morphology and the structure of RBC-NPs were visualized under a transmission electron microscopy (TEM, JEOL JEM-2010, Tokyo, Japan). The stability of RBC-NPs was investigated by detection of the particle size during storage. Briefly, RBC-NPs were stored in 1× PBS under 4 °C. The diameters of different-sized RBC-NPs were measured every day for a week.

### 2.5. Membrane Protein Analysis of RBC-NPs

Membrane proteins of RBC-NPs were analyzed by sodium dodecyl sulfate-polyacrylamide gel electrophoresis (SDS-PAGE) assay and compared with the RBC ghost. In brief, samples of 80 nm RBC-NPs were prepared in SDS sample buffer (Invitrogen), and the total protein content was quantified by the Bradford method to normalize them to an equal protein concentration (1 mg/mL). Then, protein samples were heated at 90 °C for 5 min, loaded onto the 10% SDS-polyacrylamide gel, and run at 120 V for 1 h followed by Coomassie blue staining and imaging. Western blotting was conducted to examine specific proteins on the RBC membrane, such as CD47, a typical marker of self with an immunomodulatory effect [44]. Briefly, samples were prepared with NuPAGE™ LDS sample buffer (Invitrogen), separated by SDS-PAGE, transferred to nitrocellulose membranes, and incubated with antibodies specific for CD47 overnight, followed by staining with horseradish peroxidase (HRP)-conjugated secondary antibody. Then the proteins were detected using the Immobilon™ Western Chemiluminescent HRP Substrate, and the band intensity was read by the ImageJ software (National Institutes of Health, USA, 1.48v).

The protein composition of 80 nm RBC-NPs was further investigated by label-free quantification proteomics as previously described [45]. Briefly, RBC-NPs samples were prepared in the lysis buffer containing 7.8 mM *n*-dodecyl-β-d-maltoside, 50 mM Tris-HCl, 750 mM aminocaproic acid, and 0.5 mM EDTA (pH 7.0). Afterward, the six-fold volume of acetone was added to the sample followed by incubation at −20 °C overnight. The protein pellet was then collected by centrifugation, dissolved in the buffer containing 300 mM triethylamine borane and 6 M guanidine hydrochlorides, quantified by the Bradford method, and digested by trypsin. The peptide sample was then subjected to a Nano ACQUITY UPLC system (Waters Corporation, Milford, MA) connected to an Orbitrap Fusion mass spectrometer (Thermo Fisher Scientific, Bremen, Germany). The mobile phase A was 0.1% formic acid (*v*/*v*) in water, and the mobile phase B was acetone containing 0.1% (*v*/*v*) formic acid. 5 μL of peptide sample was injected and separated on the analytical column (Acclaim PepMap C18, 75 μm × 25 cm) with a linear gradient from 5% B to 30% B in 110 min. The flow rate was 300 nL/min, and the column temperature was 45 °C. A positive electrospray voltage of 2 kV and a 300 °C capillary temperature were applied for ionization. Data-dependent tandem mass spectrometry (MS) analysis was performed using a top-speed approach with an isolation width of 2 Da. The normalized collision energy was set at 35% for higher energy collision-induced dissociation. The typical MS/MS scan conditions were as follows: a targeted automatic gain control (AGC) value of 2 × 10^5^; a maximum fill time of 100 ms; an intensity threshold of 50,000 for fragmentation. A dynamic exclusion of 24 s was applied with a mass tolerance of 10 ppm. The MS/MS fixed first mass was set at 110. The acquired MS/MS spectra were searched using MaxQuant (version 1.6.1.0, Max Planck Institute of Biochemistry, Martinsried, Germany) against the National Center for Biotechnology Information (NCBI) mouse RefSeq protein databases (updated on 04-19-2018). The iBAQ quantification (intensity-based absolute quantification) was used for label-free quantitation.

### 2.6. Cellular Uptake RBC-NPs by Macrophages

To examine the macrophage uptake of different-sized RBC-NPs, RAW 264.7 murine macrophage cells were seeded at a density of 10^5^ cells per well on 12-well plates and cultured in DMEM supplemented with 10% FBS for 24 h. Afterward, cells were incubated with coumarin-6-labeled RBC-NPs with different sizes (0.5 mg/mL) at 37 °C for 2 h and then washed with PBS for the fluorescence imaging or the flow cytometry assay. For the fluorescence imaging, cells were fixed with 4% paraformaldehyde at room temperature for 15 min, stained with DAPI for 5 min, and finally observed by a laser scanning confocal microscope (ZEISS, 710, LSM, Jena, Germany). Fluorescent images were captured in the two channels relevant to DAPI and coumarin-6. To quantify the cellular uptake of RBC-NPs by macrophage cells, cells were harvested by trypsin digestion, suspended in PBS, and then subjected to the flow cytometry (BD FACSAria II, MD, USA) for the measurement of fluorescence intensity of cells.

### 2.7. Filtration Test of RBC-NPs

To investigate the ability of RBC-NPs to pass through filter membranes, different-sized RBC-NPs were extruded through 0.10 μm and 0.22 μm syringe filters with cellulose acetate membranes, respectively. The turbidity of RBC-NPs before and after filtration was measured at 560 nm and the relative retention of RBC-NPs in the filtrate was calculated by dividing the turbidity of RBC-NPs after filtration by the turbidity of RBC-NPs before filtration. The hydrodynamic size of RBC-NPs in the filtrate was tested by a DLS detector (Zetasizer, Nano ZS, Malvern, UK).

### 2.8. Pharmacokinetics and Biodistribution of RBC-NPs

For the pharmacokinetics study, ICR mice were randomly divided into four groups (*n* = 6) and respectively injected with 150 μL of differently sized DiD-labelled RBC-NPs in PBS at a dose of 30 mg/kg of PLGA via the tail vein. 50 μL of blood was collected by cheek pouch puncture at 1 min, 5 min, 10 min, 30 min, 1 h, 3 h, 8 h, 24 h, and 48 h after injection, respectively. The fluorescence intensity of blood samples was quantified by a Tecan Infinite M200 Pro Multiplate Reader (Mannedorf, Switzerland) with an excitation wavelength of 640 nm and an emission wavelength of 670 nm. The concentration of RBC-NPs in the blood was expressed as the percentage of injected dose per milliliter (% ID/mL). Pharmacokinetics parameters, including area under the concentration-time curve (*AUC_0–t_*), mean residence time (*MRT_0–t_*), elimination rate constant (*k*), clearance (*Cl*), and the half-life (*t_1/2_*), were calculated by DAS 3.0 pharmacokinetics software (BioGuider Co., Shanghai, China).

For the biodistribution study, ICR mice were injected with 150 μL of differently sized DiD-labelled RBC-NPs in PBS at a dose of 30 mg/kg of PLGA via the tail vein. Forty-eight hours after injection, mice were sacrificed followed by heart perfusion with saline, and major organs including the heart, the liver, the spleen, the lung, the kidney, the brain, and the blood were collected. After tissue homogenization with saline, the fluorescence intensity of tissue samples was quantified by a Tecan Infinite M200 Pro Multiplate Reader (Switzerland) with an excitation wavelength of 640 nm and an emission wavelength of 670 nm. The concentration of RBC-NPs in tissues was expressed as the percentage of injected dose per gram of tissue (% ID/g).

To observe the distribution of RBC-NPs in major organs, mice were injected 150 μL of differently sized DiD-labelled RBC-NPs in PBS at a dose of 30 mg/kg of PLGA via the tail vein. After 48 h, mice were sacrificed followed by heart perfusion with saline, and then major organs including the heart, the liver, the spleen, the lung, the kidney, and the brain were collected. These organs were fixed in 4% paraformaldehyde for 24 h, dehydrated in a 30% sucrose solution, embedded in Tissue Tek^®^ opti-mum cutting temperature (O.T.C.) compound (Sakura, USA), and sliced into frozen sections of 10 μm thickness for subsequent immunostaining. The sections were successively labeled with CD31 goat polyclonal primary antibody, Alexa Fluor^®^ 488-conjugated donkey-goat secondary antibody, and DAPI. Then the sections were observed under a laser scanning confocal microscope (ZEISS, 710, LSM, Germany). To semi-quantify the distribution of RBC-NPs in these tissues, the fluorescence intensity of RBC-NPs in organ slices from six randomly assigned regions in each organ (*n* = 6) was quantified by the ZEN 2012 software at 120× magnification.

### 2.9. Immunogenic Response

The immunogenic response of different-sized RBC-NPs was investigated by measuring the immunoglobulin M (IgM) and immunoglobulin G (IgG) levels in serum from the mice injected with RBC-NPs. In brief, ICR mice were randomly divided into five groups (*n* = 5 per group). Mice received an injection of different-sized RBC-NPs at a dose of 30 mg/kg of PLGA, respectively, except that mice in the control group received an injection of an equal volume of saline. Five days after injection, 1 mL of blood was collected from each mouse through orbital sinus and serum was prepared for IgM and IgG measurement according to the ELISA protocol.

### 2.10. Statistical Analysis

Data were expressed as mean ± standard deviation (SD). One-way analysis of variance (ANOVA) followed by Tukey’s post hoc test was used to determine differences between groups. The unpaired student’s *t*-test was used for the assessment of statistically significant differences between the two groups. *p* values < 0.05 were considered statistically significant.

## 3. Results and Discussions

### 3.1. Characterization and Stability of RBC-NPs

**Figure 1:**
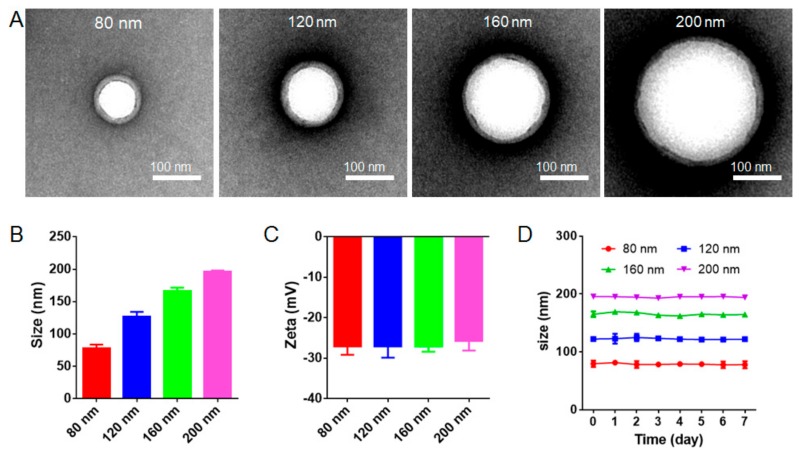
Characterization of red blood cell membrane-coated nanoparticles (RBC-NPs) with different diameters. (**A**) Transmission electron microscopy (TEM) images of RBC-NPs with diameters of 80 nm, 120 nm, 160 nm, and 200 nm demonstrated the core/shell structure of RBC-NPs. Scale bar = 100 nm. (**B**) Size and (**C**) surface zeta potential of RBC-NPs with four different diameters (*n* = 3). (**D**) Diameter-dependent stability of different-sized RBC-NPs at 4 °C for 7 days (*n* = 3). All error bars represent standard error of the mean.

The preparation of RBC-NPs included the following steps: extracting RBC membranes, preparing PLGA nanoparticle cores, and fusing RBC membranes onto the surface of PLGA nanoparticle cores by sonication. For the preparation of PLGA nanoparticle cores, the diameters of nanoparticle cores increased with the PLGA concentration. After the RBC membrane coating on nanoparticles, the white spherical PLGA nanoparticle cores and the outer RBC membrane rings were clearly observed under TEM (Figure 1A and Figure S1), which suggested that PLGA nanoparticle cores were successfully coated by RBC membranes. The thickness of RBC membranes on differently sized nanoparticles was uniformly around 8 nm, which agreed well with previous results [7]. The DLS results indicated that the diameters of four kinds of resulting RBC-NPs were approximately 80 nm, 120 nm, 160 nm, and 200 nm with a relatively low polydispersity index, respectively (Figure 1B and Figure S2). There was no significant difference in the surface zeta potential of differently sized RBC-NPs (Figure 1C), and their zeta potentials were similar to RBC membrane vesicles (about −27 mV), indicating again that PLGA nanoparticle cores were successfully coated with RBC membranes. After keeping four different-sized RBC-NPs at 4 °C for 7 days (Figure 1D), the DLS results showed that all RBC-NPs had a good stability, and the particle size did not influence the stability of RBC-NPs.

**Figure 2:**
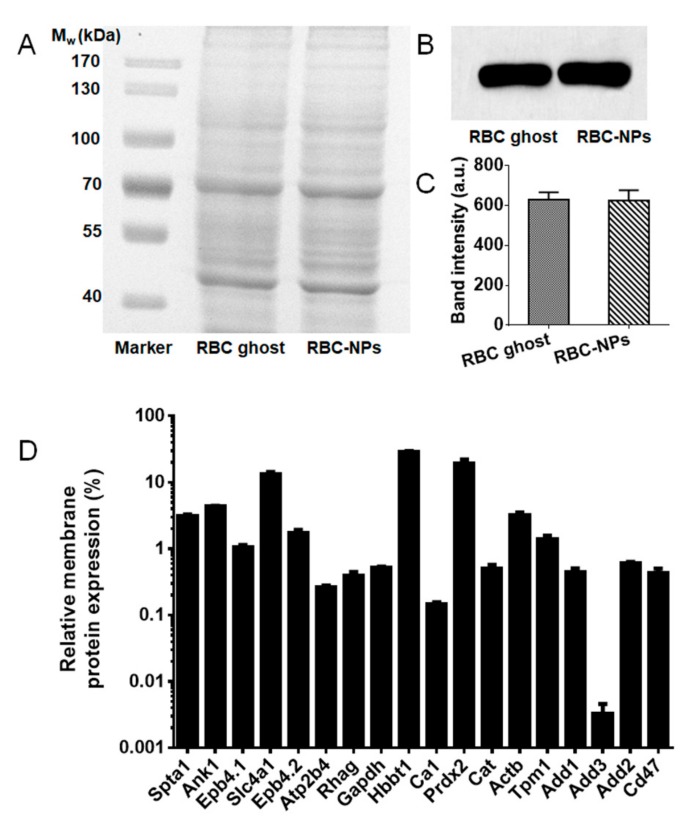
Protein characterization. (**A**) SDS-PAGE protein analysis of RBC ghost and RBC-NPs at equivalent protein concentrations. (**B**) Western blotting analysis of membrane-specific protein marker CD47 in RBC ghost and RBC-NPs. (**C**) Quantification of western blot band intensity of CD47 in RBC ghost and RBC-NPs (*n* = 3). (**D**) The relative expression of typical RBC membrane proteins based on the label-free quantification proteomics.

The membrane protein retention on RBC-NPs was determined by SDS-PAGE using 80 nm RBC-NPs as model biomimetic nanoparticles. As shown in Figure 2A, RBC-NPs retained almost all of the cell membrane proteins compared with the RBC ghost. Additionally, further western blot analysis revealed that the RBC ghost and RBC-NPs had similar blotting patterns (Figure 2B). Quantification of western blot band intensity showed that the RBC ghost and RBC-NPs contained equivalent amounts of CD47 proteins (Figure 2C), indicating that the key protein CD47 was present on RBC-NPs without loss after the coating process. The protein composition of 80 nm RBC-NPs as model biomimetic nanoparticles was further investigated by the label-free quantification proteomics. In RBC-NPs, 474 mouse membrane proteins were identified. These proteins were classified according to the biological process: transport (83), proteolysis (36), oxidation-reduction process (36), protein folding (34), protein transport (33), metabolic process (25), response to drug (24), ubiquitin-dependent protein catabolic process (23), negative regulation of apoptotic process (23), ion transport (23) protein stabilization (21), cell-cell adhesion (21), and others. Further quantitative analysis of these proteins including calcium-transporting ATPase (gene: *Atp2b4*), ammonium transporter Rh type A (gene: *Rhag*), Glyceraldehyde-3-phosphate dehydrogenase (gene: *Gapdh*), cytoskeletal proteins (e.g., spectrin (gene: *Spta1*), ankyrin 1 (gene: *Ank1*), β-adducin (gene: *Add2*), α-adducin (gene: *Add1*), protein 4.1 (gene: *Epb4.1*), mouse-specific gamma-adducin (gene: *Add3*)), and leukocyte surface antigen CD47 revealed that RBC-NPs retained an abundant expression of RBC membrane proteins (Figure 2D). Notably, RBC-NPs exhibited a high expression of CD47, which accounted for 0.423% of the total membrane proteins.

### 3.2. Cellular Uptake of RBC-NPs by Macrophages

**Figure 3:**
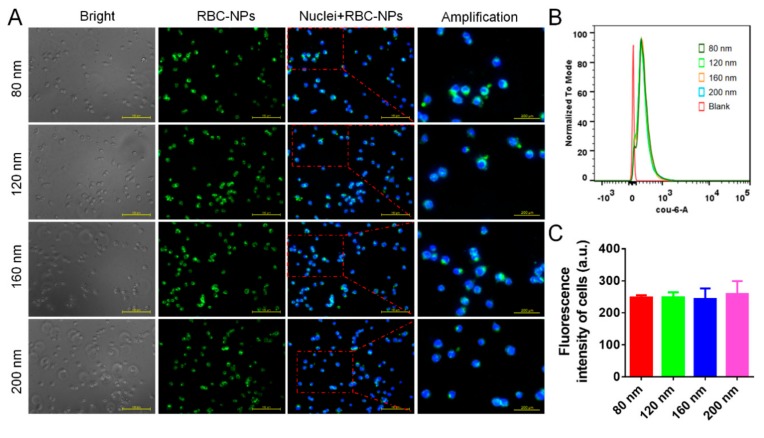
Cellular uptake experiment. (**A**) Fluorescence images of cellular uptake of four different-sized RBC-NPs by RAW 264.7 cells. (**B**) Flow cytometry and (**C**) corresponding quantitative results of cellular uptake of four different-sized RBC-NPs by RAW 264.7 cells (*n* = 4).

Macrophages are important cells responsible for the in vivo immune clearance of nanoparticles. The phagocytosis of macrophages is regulated by two factors: “eat me” signal produced by antigens, and “do not eat me” signal produced by immunosuppressive substances. CD47 proteins, the “do not eat me” signal on the surface of healthy RBCs, interact with signal regulatory protein α (SIRPα), a regulatory membrane glycoprotein from the SIRP family expressed by macrophages, and inhibit the host cell phagocytosis of macrophages [46]. When RBCs senesce and are near to death, CD47 proteins reduce greatly and the “do not eat me” signal disappears. Valgus phosphatidylserine produces an “eat me” signal to attract macrophages, and then immune cells start to clear them [47,48,49]. Considering that RBC membranes from the fresh blood coat PLGA nanoparticles in the right-side-out direction [42], RBC-NPs have surface CD47 proteins similar to those of healthy RBCs, thus escaping from the phagocytosis of macrophages. It was verified that four different-sized RBC-NPs have a similarly low uptake by macrophages (Figure 3A), and no significance in the cellular uptake was found in different groups (Figure 3B,C). These results suggested that the cellular uptake of RBC-NPs was not determined by the size but by was determined by their surface CD47 proteins.

### 3.3. Filtration through Filter Membranes

**Figure 4:**
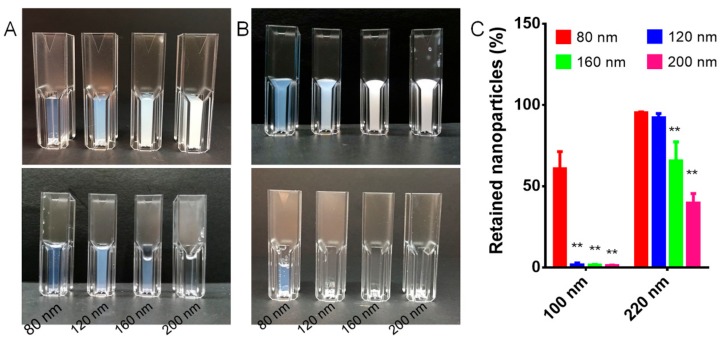
Photo images different-sized RBC-NPs before (upper row) and after (lower row) passing through (**A**) 0.22 μm or (**B**) 0.1 μm syringe filters. (**C**) The relative retention of RBC-NPs in the filtrate after passing through 0.22 μm or 0.1 μm syringe filters. ** *p* < 0.01 compared with 80 nm RBC-NPs.

RBCs are disk-shaped, and the RBC membrane is a lipid bilayer structure, under which there are spectrins. RBC membranes have a flexible surface, and the cytoplasm is mobile, which can rearrange the cytoskeleton and make RBCs be extruded to pass the capillary smaller than RBCs. However, the PLGA cores in the RBC-NPs are small hard entities, so they might not pass through some in vivo biological barriers easily. The liver and the spleen are major biological barriers for nanomedicine delivery because they sequester most of the administered nanomedicines and prevent their delivery to target sites [50]. The physiologic pore size of the hepatic sinusoidal capillaries for nanoparticle filtration varies among different mammalian species. The capillary fenestrae of human hepatic sinusoids are on average approximately 105 nm in diameter, while those of rodent hepatic sinusoids are on average approximately 135 nm [39,51]. The spleen can also filter nanoparticles. It has been reported that the slit size in the spleen rarely exceeds 200 to 500 nm in width [40]. Therefore, syringe filters with cellulose acetate membranes having a pore size of 0.22 μm and 0.1 μm were used to mimic the liver and the spleen filtration and test the ability of hard RBC-NPs to pass through slits narrower than their sizes. The cellulose acetate membrane is hydrophilic and does not adsorb RBC-NPs on its surface. As shown in Figure 4, RBC-NPs bigger than 120 nm (Figure 4A–C) could not pass through 0.1 μm syringe filters but partly pass through 0.22 μm syringe filters. Eighty nm RBC-NPs seemed to pass through 0.22 μm syringe filters unrestrictedly but partly pass through 0.1 μm syringe filters. The turbidimetric assay showed that nearly 100% and 62% of 80 nm RBC-NPs remained in the filtrate after passage through 0.22 μm and 0.1 μm syringe filters, respectively. The hydrodynamic size of RBC-NPs after filtration through 0.22 μm syringe filters was also tested. No change could be observed in the 80 nm RBC-NPs group, indicating the RBC-NPs remained intact during filtration. However, the hydrodynamic size of RBC-NPs bigger than 160 nm was sharply reduced. These results suggested that hard RBC-NPs could not pass through narrow slits smaller than their size, and they might be trapped in the liver and the spleen if their size was not small enough.

### 3.4. Pharmacokinetics and Biodistribution

**Figure 5:**
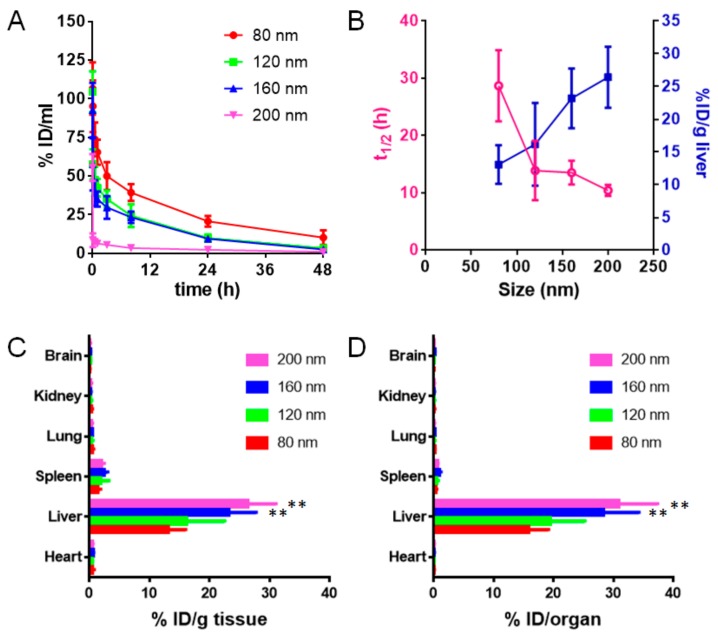
In vivo pharmacokinetics and tissue distribution of RBC-NPs. (**A**) In vivo pharmacokinetic curve of four different-sized RBC-NPs. (**B**) The correlation between *t_1/2_* (h) and size (nm) of RBC-NPs or the correlation between liver distribution (% ID/g liver) and size (nm) of RBC-NPs. (**C**) Tissue distribution (% ID/g tissue) of four different-sized RBC-NPs 48 h post intravenous injection. (**D**) Tissue distribution (% ID/organ) of four different-sized RBC-NPs 48 h post intravenous injection. ** *p* < 0.01 compared with 80 nm.

**Figure 6:**
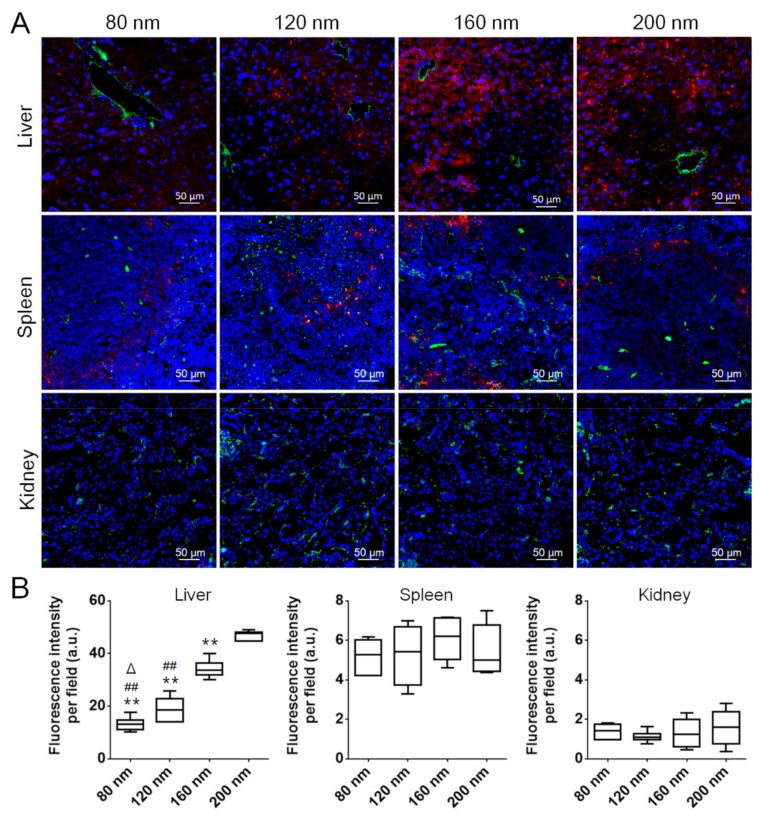
RBC-NP distribution in organ slices 48 h post intravenous injection. (**A**) Confocal microscopy images of four different-sized RBC-NPs in liver, spleen and kidney slices. Red represents RBC-NPs. Blue represents cell nuclei. Green represents blood vessels. (**B**) Fluorescence intensity of four different-sized RBC-NPs in liver, spleen, and kidney slices. ** *p* < 0.01 compared with 200 nm. ^##^ *p* < 0.01 compared with 160 nm. ^Δ^ *p* < 0.01 compared with 120 nm.

The life span of RBCs is about four months (120 days), which is much longer than the in vivo life of most nanoparticles and inspires to make RBC membrane-coated nanoparticles avoiding the clearance of immune cells. Although RBC membrane-coated nanoparticles have a longer circulation than PEGylated nanoparticles [7], they are also easily cleared in vivo and mainly accumulated in the mononuclear phagocyte system (MPS)-related organs such as the liver and the spleen, similar to PEGylated nanoparticles. It was shown that the circulation life and biodistribution of PEGylated nanoparticles were size-dependent [1]. 100 nm PEGylated nanoparticles have an optimal circulation life, and larger or smaller nanoparticles have a shorter circulation life than 100 nm PEGylated nanoparticles. Our results also showed that particle size significantly influenced the in vivo pharmacokinetics of RBC-NPs. As the particle size became smaller, the in vivo elimination time of RBC-NPs was prolonged (Figure 5A), and the in vivo half-life (*t_1/2_*) was prolonged (Figure 5B). The biodistribution results showed that four different-sized RBC-NPs were all mainly distributed in the liver, followed by the spleen, the lung, the kidney, the heart, and the brain (Figure 5C,D). The larger the particle size of RBC-NPs was, the more they tended to accumulate in the liver (Figure 5B–D). Further observation of RBC-NPs in organ slices revealed that all RBC-NPs were mainly distributed in the liver and the spleen (Figure 6A,B). Their distribution in the liver increased with their size, which agreed well with the biodistribution results. These results indicated that the particle size of RBC-NPs had a great influence on their pharmacokinetics and tissue distribution.

Recently, it has been found that the liver flow dynamics and cellular phenotype contribute greatly to the accumulation of conventional nanomaterials with different surface chemistries [50]. As nanomaterials enter the liver, their velocity is sharply reduced (1000-fold), leading to more nanomaterial interaction with hepatic cells relative to peripheral cells. Kupffer cells (similar to macrophages), hepatic B cells, and sinusoidal endothelial cells mainly contribute to the uptake of nanomaterials in the liver [50]. Intriguingly, in this study, sinusoidal endothelial cells showed low uptake of RBC-NPs as little overlay of RBC-NPs with sinusoidal endothelial cells was found in all groups (Figure 6A), indicating the distribution pattern of RBC-NPs in the liver was different from that of conventional nanoparticles. Considering that the particle size did not influence the cellular uptake of RBC-NPs by macrophages, the size-dependent circulation and distribution in the liver might be mainly due to the liver filtration of RBC-NPs.

It has been shown that the physicochemical properties of nanoparticles, such as shape [35], size [52,53], surface chemistry [54], elasticity [55], topographical structure [56], and crystallization [57], determine their absorption of serum proteins, cellular uptake by macrophages, pharmacokinetics, and biodistribution in vivo. For instance, the serum protein adsorption and phagocytic uptake are markedly decreased for the nanoparticles with higher PEG length or smaller size [52]. The conjugation of a second PEG layer at a density close to but lower than the mushroom-to-brush transition regime on conventional PEGylated nanoparticles dramatically prolongs their blood circulation via reduced nanoparticle uptake by non-Kupffer cells in the liver, especially liver sinusoidal endothelial cells [56]. Softer nanoparticles exhibit significantly reduced cellular uptake by immune cells, endothelial cells, and cancer cells, and they offer enhanced circulation and subsequently enhanced targeting compared with harder nanoparticles in vivo [55]. It is observed that softer Zwitterionic nanogels pass through physiological barriers, especially the splenic filtration, more easily than their stiffer counterparts, consequently leading to a longer circulation half-life and lower splenic accumulation [40]. However, in the present study, reducing the size of hard biomimetic nanoparticles could improve the passage of nanoparticles across the liver sinusoids, decrease the trapped nanoparticles, and thus extend the circulation life of biomimetic nanoparticles.

### 3.5. Immunogenic Response

**Figure 7:**
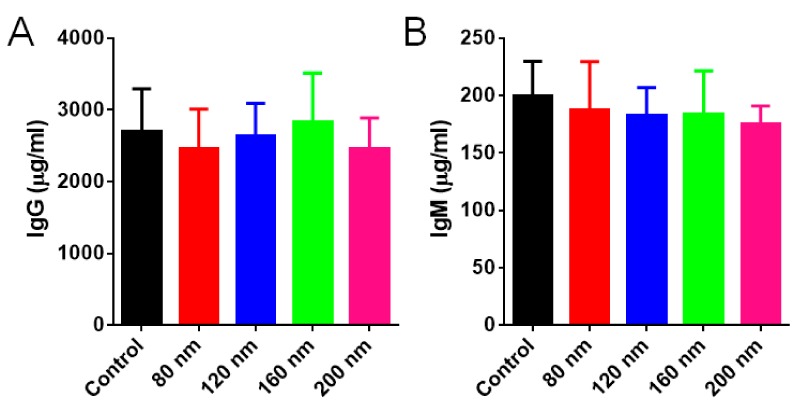
IgG and IgM detection. (**A**) The serum content of IgG (μg/mL) of mice treated with four different-sized RBC-NPs. (**B**) The serum content of IgM (μg/mL) of mice treated with four different-sized RBC-NPs.

From the aspect of IgG and IgM detection, it was found there were no significant differences in the serum IgG or IgM level in all groups (Figure 7A,B), indicating that different-sized RBC-NPs did not cause significant immunogenic responses. The results were well consistent with previous reports [58], which might owe to the surface coating of RBC membranes on PLGA nanoparticles. The RBC membrane not only provides PLGA nanoparticles a dense bilayered lipid barrier against the recognition of immune cells but also contains numerous surface antigens such as CD47 (Figure 2D) that can interact with or even help escape from the monitoring of immune cells.

## 4. Conclusions

To find out some clues determining the in vivo life of biomimetic nanoparticles, RBC-NPs was selected as the model biomimetic nanoparticles and the influence of particle size on the cellular uptake by macrophages, filtration through syringe filters, circulation, biodistribution, and immunological response of RBC-NPs were investigated. It was found that the cellular uptake of RBC-NPs by macrophage cells in vitro and their immunogenic responses in vivo were not influenced by the particle size. However, their circulation life in vivo decreased with the particle size while the nanoparticle accumulation in the liver increased with the particle size, which might be related to their size-dependent filtration through hepatic sinusoids. The findings will provide experimental evidence for the design and the optimization of biomimetic nanoparticles.

## Figures and Tables

**Table 1:** Red blood cell (RBC) membranes required to fully coat differently sized poly(lactic-co-glycolic acid) (PLGA) cores.

Groups	Core Size (nm)	Surface Area Per Core (μm^2^)	Mass Per Core (×10^−9^ μg)	Surface Area Per Milligram of Core (×10^10^ μm^2^)	RBC Ghost Required to Coat 1 Milligram of Core (μL)
0 nm	60	0.0113	0.136	8.33	138.9
120 nm	100	0.0314	0.628	5.00	83.4
160 nm	140	0.0615	1.72	3.57	59.6
200 nm	160	0.0804	2.57	3.13	52.1

## Supplementary Materials

The following are available online at https://www.mdpi.com/2073-4409/8/8/881/s1, Figure S1: TEM images of RBC-NPs with diameters of 80 nm, 120 nm, 160 nm and 200 nm. S2: Polydispersity index of RBC-NPs with four different diameters (*n* = 3).
