# Supplementary material for: Size Dependency of Circulation and Biodistribution of Biomimetic Nanoparticles: Red Blood Cell Membrane-Coated Nanoparticles
Source: Cells. 2019 Aug 13;8(8):881. doi: 10.3390/cells8080881 (PMC6721642; doi:10.3390/cells8080881)
Supplement: Supplementary file 1 [file cells-08-00881-s001.pdf]

Supporting materials

Size dependency of circulation and biodistribution of biomimetic nanoparticles: red blood cell membrane-coated nanoparticles

Haichun Li, Kai Jin, Man Luo, Xuejun Wang, Xiaowen Zhu, Xianping Liu, Ting Jiang, Qin Zhang, Zhiqing Pang* and Sheng Wang*

* Correspondence: zqpang@fudan.edu.cn; Tel.: +86-21-51980067; author initials: Z. Pang; wangs601@163.com; author initials: S. Wang

Results

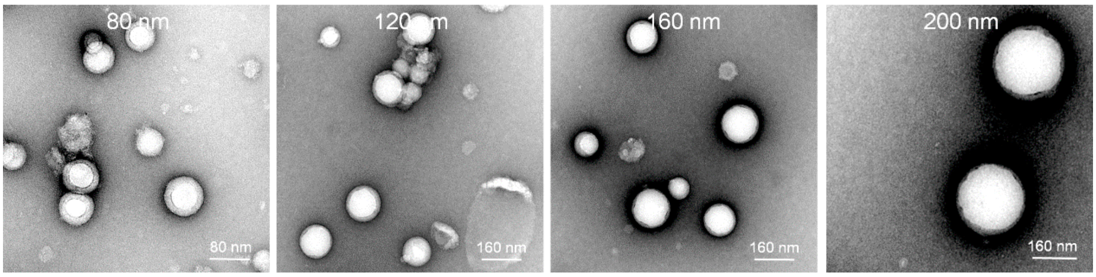

Figure S1 TEM images of RBC-NPs with diameters of 80 nm, 120 nm, 160 nm and 200 nm.

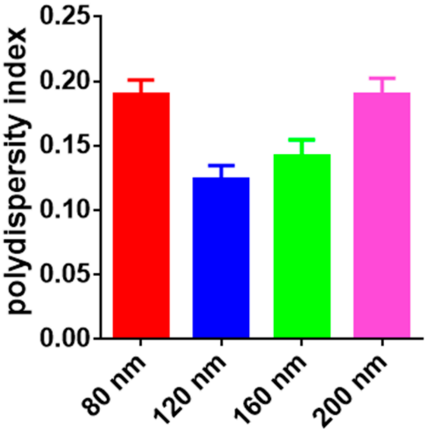

Figure S2 Polydispersity index of RBC-NPs with four different diameters (n=3).
